# Application of the INFOGEST Standardized Method to Assess the Digestive Stability and Bioaccessibility of Phenolic Compounds from Galician Extra-Virgin Olive Oil

**DOI:** 10.1021/acs.jafc.1c04592

**Published:** 2021-09-22

**Authors:** P. Reboredo-Rodríguez, L. Olmo-García, M. Figueiredo-González, C. González-Barreiro, A. Carrasco-Pancorbo, B. Cancho-Grande

**Affiliations:** † Food and Health Omics, Department of Analytical and Food Chemistry, Faculty of Science, University of Vigo, 32004 Ourense, Spain; ‡ Department of Analytical Chemistry, Faculty of Science, University of Granada, Ave. Fuentenueva s/n, 18071 Granada, Spain

**Keywords:** extra-virgin olive oil, phenolic compounds, antioxidant capacity, *in vitro* digestion, bioaccessibility, α-glucosidase inhibition

## Abstract

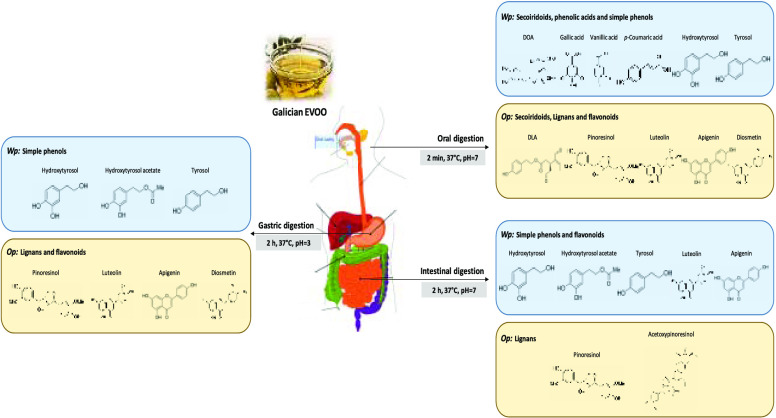

The INFOGEST standardized method was applied to assess the potential bioaccessibility and bioaccessibility of the phenolic compounds from a Galician extra-virgin olive oil (EVOO). The *in vitro* digestion model involves three steps and generates two fractions after each one: an aqueous fraction (namely, water phase (Wp)) and an oily fraction (namely, oily phase (Op)). The results showed that secoiridoids were the most abundant family in the Galician EVOO polar fraction, representing 98% of the total phenolic compounds. After oral digestion, phenolic acids and simple phenols were mainly detected in Wp, while lignans and flavonoids were mostly found in Op. After gastric digestion, extensive hydrolysis of secoiridoids was observed to generate free tyrosol, hydroxytyrosol, and hydroxytyrosol acetate. The instability of secoiridoids after intestinal digestion was again responsible for the release of simple phenols, which were mainly recovered in Wp together with flavonoids. In contrast, lignans were stable to duodenal conditions and remained in Op.

## Introduction

The Mediterranean diet (MD), which is characterized by a high intake of exogenous dietary phenolics as a consequence of daily consumption of vegetables, fruits, nuts, whole grains, and healthy heats, has been associated with a lower incidence of several diseases.^1−3^ Virgin olive oil (VOO) is the primary source of added fat in the MD. It provides monounsaturated fat, which has been found to reduce total cholesterol and low-density lipoprotein (LDL) cholesterol levels. VOO is also valued for the health benefits attributed to its phenolic compounds, whose occurrence depends on many factors, one of the most important being the olive variety. Hydroxytyrosol (HTy) and tyrosol (Ty) together with their secoiridoid derivatives are the most representative phenols in VOO. In addition, lignans such as pinoresinol (Pin) and acetoxypinoresinol (Ac-Pin), flavonoids like luteolin (Lut) and apigenin (Api), and phenolic acids such as *p*-coumaric (*p*-Cou) and vanillic acid (Van) can also be found to a minor extent.^4^

Galicia (N.W. Spain) has gradually emerged as a promising olive-growing region producing high-quality and distinctive extra-virgin olive oils (EVOOs). Galician EVOOs obtained from old autochthonous varieties, “Brava Gallega” and “Mansa de Figueiredo”, are characterized by their high content on phenolic compounds.^5,6^ Figueiredo-González et al. evaluated the role of dietary polyphenols from these EVOOs against the inhibition of key enzymes involved in the management of type 2 diabetes (α-glucosidase and α-amylase).^7^ Their findings support the potential health benefits derived from Galician EVOOs, which might be linked to their outstanding concentration levels of phenolic acids and flavonoids.

The biological effects of EVOO bioactive phenolics are conditioned by their bioaccessibility, bioavailability, and metabolic fate. Bioaccessibility, the first requirement, is defined as the amount of phenolic compounds extracted from the EVOO matrix that might be able to pass through the intestinal barrier.^8,9^ Bioavailability, the second requirement, is related to the portion of phenolic compounds that is digested, absorbed, and metabolized through the normal pathways.^8,9^ Since the bioavailability of bioactive phenolics depends on their digestive stability, their release from the oil matrix and the efficiency of their trans epithelial passage should be investigated.

The *in vitro* static methods simulating gastrointestinal digestion have been very useful to predict bioaccessibility and bioavailability. To date, there are a few publications focused on the bioaccessibility and stability of phenolic compounds from EVOOs during *in vitro* digestion. Some of these studies applied combined models of simulated digestion and cell culture markers to assess the stability and antioxidant activities of oils after *in vitro* digestion by Folin–Ciocalteu, 2,2-diphenyl-1-picrylhydrazyl (DPPH), 2,2′-azino-bis(3-ethylbenzothiazoline-6-sulfonic acid) (ABTS), and ferric reducing antioxidant power (FRAP) methods.^8,10−12^ Pereira-Caro and co-workers used liquid chromatography with diode array detection (LC–DAD) to evaluate the digestive stability of HTy, HTy acetate (HTy-Ac), and alkyl HTy esters.^13^ Recently, LC coupled to mass spectrometry (LC-MS) was applied to the analysis of phenolic compounds to evaluate the transformations of EVOO antioxidants during the gastrointestinal process.^9,14,15^

When experimental conditions of the above *in vitro* methods were compared, important variations were detected in experimental parameters. This fact impedes the meaningful comparison of published results. To reach a consensus on some digestion parameters for static *in vitro* simulation of an adult digestion, the international INFOGEST network has recently published a standardized method. Using this method, food samples are subjected to sequential oral, gastric, and intestinal digestion while parameters such as electrolytes, enzymes, bile, dilution, pH, and time of digestion are based on available physiological data.^16^

Taking this into account, the first aim of the present study was to characterize the phenolic composition of a commercial EVOO obtained by co-crushing Galician “Brava Gallega” and “Mansa de Figueiredo” old autochthonous varieties. The second goal of this work was to evaluate the digestive stability of its phenolic compounds using the INFOGEST standardized *in vitro* gastrointestinal method and a membrane dialysis system. We proposed to incorporate a dialysis membrane during intestinal digestion to provide a reliable estimation of phenolics bioaccessibility. Stability and antioxidant capacities (AC) of the phenolic fraction before and after *in vitro* digestion were studied by Folin–Ciocalteu and DPPH methods; transformations of phenolic compounds were evaluated using LC with several detectors: DAD, fluorescence (FLD) and MS, the latter combining the use of tandem mass spectrometry (MS/MS) and high-resolution mass analyzers. Furthermore, the third objective of this study was to evaluate the involvement of the phenolic compounds from the selected Galician EVOO and its resulting bioaccessible fraction (Bf) against the inhibition of α-glucosidase.

## Materials and Methods

### Chemicals and Reagents

#### Analysis of Phenolic Compounds

Methanol (MeOH) and acetonitrile (ACN) LC-MS grade were acquired from Prolabo (Paris, France). Deionized water was obtained using a Milli-Q system from Millipore (Bedford, MA). Ethanol (EtOH) HPLC PLUS Gradient grade was purchased from Carlo Erba Reagents (Barcelona, Spain), and *n*-hexane for HPLC (≥97.0%) was obtained from Honeywell (Muskegon, MI). Acetic acid (AcH), Folin–Ciocalteu reagent, sodium carbonate (Na_2_CO_3_), gallic acid (GA), Trolox, and DPPH were acquired from Sigma-Aldrich (St. Louis, MO), and sodium molybdate dihydrate (Na_2_MoO_4_·2H_2_O) was provided by Scharlab (Barcelona, Spain).

Commercially available pure standards were acquired both for qualitative and quantitative purposes. HTy, Ty, Lut, Pin, Api, GA, *p*-Cou, ferulic acid (Fer), and Van were purchased from Sigma-Aldrich, and oleuropein (Ole) was delivered by Extrasynthese (Lyon, France). Stock solutions for each analyte were prepared by dissolving the appropriate amount of each chemical standard in ACN/H_2_O (50:50, v/v). After that, they were serially diluted to prepare the working solutions which covered concentration levels over the range from the quantification limit to 250 mg/L. 3,4-Dihydroxyphenylacetic acid (DOPAC), acquired from Sigma-Aldrich, was used as an internal standard (IS).

OASIS HLB 6 cc (200 mg) solid-phase extraction (SPE) cartridges were supplied by Waters Corp. (Milford, MA), and 0.22 μm poly(vinylidene difluoride) (PVDF) syringe filters were provided by Scharlab.

#### *In Vitro* Digestion Assays

Pepsin from porcine gastric mucosa, bile extract porcine, and pancreatin from porcine pancreas were purchased from Sigma-Aldrich; rabbit gastric extract was acquired from Lipolytech (Marseille, France). Calcium chloride (CaCl_2_(H_2_O)_2_, 96%) and hydrochloric acid (HCl, 37%) were provided by Scharlab. Ammonium carbonate ((NH_4_)_2_CO_3_, 30–34% in NH_3_), potassium chloride (KCl, 99%) sodium bicarbonate (NaHCO_3_, 99–101%), and sodium chloride (NaCl, 99.5%) were purchased from Panreac (Barcelona, Spain). Monopotassium phosphate (KH_2_PO_4_, ≥99.0%) was provided by Sigma-Aldrich, and magnesium chloride hexahydrate (MgCl_2_(H_2_0)_6_, 99.0–101.0%) was obtained from Merck (Darmstadt, Germany). Dialysis membrane tubing (12 000–14 000 Da) MWCO was supplied by Spectrum Laboratories, Inc. (Piscataway, NJ).

#### *In Vitro* Enzyme Inhibition Assays

α-Glucosidase (=maltase from *Saccharomyces cerevisiae*), 4-nitrophenyl α-d-glucopyranoside (PNP-G), potassium dihydrogen phosphate (H_2_KPO_4_), and sodium hydroxide (NaOH) were obtained from Sigma-Aldrich.

### EVOO Sample

A Galician EVOO obtained as a result of milling together “Brava Gallega” and “Mansa de Figueiredo” olives purchased from Aceites Figueiredo S.L. (Lugo, Spain) was selected for this study. Both cultivars were produced in Ribas do Sil (Lugo, Spain) in the crop season 2019/2020. Once in the laboratory, eight 500 mL bottles were pooled and homogenized to obtain a final representative sample. Several aliquots were stored in glass amber bottles without headspace in the dark at −20 °C until use.

The Galician OO was classified as extra-virgin olive oil in accordance with the Commission Regulation (EEC) No. 2568/91 and subsequent amendments since their quality and purity indices fell within the legally established ranges (Table S1, Supporting Information).^17^

### Simulated *In Vitro* Gastrointestinal Digestion (SGD)

#### SGD Conditions

*In vitro* digestion of the Galician EVOO was performed using the recently updated harmonized INFOGEST method.^16^ Briefly, the EVOO was exposed to simulated oral, gastric, and intestinal phases containing the appropriate gastrointestinal tract components, pH values, stirring rates (55 rpm), incubation times, and temperature (37 °C). The EVOO sample (5 g) and the simulated salivary fluid (SSF, 5 mL) were added to a conical centrifuge tube (50 mL) and stirred for 5 min of incubation at pH = 7 (oral digestion). Next, the simulated gastric fluid (SGF, 10 mL) containing pepsin (2000 U/mL) and gastric lipase (60 U/mL) was added to the previous mixture and stirred for 2 h of incubation at pH = 3 (gastric digestion). Finally, the simulated intestinal fluid (SIF, 20 mL) containing bile salts (10 mM) and pancreatin (100 U/mL) was added to the previous mixture and stirred for 2 h of incubation at pH = 7 (intestinal digestion). The composition of SSF, SGF, and SIF fluids is summarized in the INFOGEST method (see Table 3 in Brodkorb et al.^16^).

To assess the bioaccessibility of the phenolic compounds, a dialysis membrane filled with NaCl (9 mg/mL, 15 mL) was placed inside the conical centrifuge tube in the last phase of the intestinal digestion.

At the end of each digestion step (oral, gastric, and intestinal), the obtained mixtures were centrifuged for 10 min at 9000 rpm (9056*g*) to separate the water phase (Wp) and the oily phase (Op). The Bf, which contained the phenolic compounds able to cross the synthetic membrane, was obtained at the end of the intestinal digestion.

#### Control Blanks in SGD

Blanks at different stages of digestion were initially prepared and analyzed by the Folin–Ciocalteau method. As expected, the blank containing SSF (an inorganic solution) for oral digestion did not reduce the Folin–Ciocalteu reagent. In the gastric digestion, the SGF is also an acidic inorganic solution that did not generate any signal at 760 nm; however, it was important to evaluate the possible interferences associated with the added gastric enzymes to obtain reliable and reproducible results. To this end, three blanks were separately evaluated: (i) SGF with pepsin did not generate any signal in the ultraviolet–visible (UV–vis) spectrophotometer; (ii) SGF with lipase generated a signal at 760 nm, and (iii) SGF with pepsin and lipase also produced the same signal. These interfering signals were reduced by 85–93% when both aqueous extracts were purified by the SPE procedure described in the Extraction of Phenolic Compounds Section. Finally, in the intestinal digestion, the SIF is an inorganic solution that did not generate any signal, but the interferences associated with bile salts and pancreatin had to be assessed too. The following three blanks were individually assessed: (i) SIF with pancreatin generated an interfering signal that disappeared by the SPE clean-up; (ii) SIF with bile salts produced a signal which was reduced by 88–90% with the SPE procedure; and (iii) SIF with pancreatin and bile salts generated an interfering signal at 760 nm that was reduced by 80–99% with the SPE procedure. It was therefore necessary to carry out a blank of the gastric and intestinal digestions in parallel to the EVOO digestion to be able to subtract the contribution of the enzymes and bile salts signal.

SPE-purified blanks were also analyzed by LC–DAD/FLD/MS to ensure the absence of interfering peaks that could compromise phenolic compounds quantification.

### Phenolic Compounds Analysis

#### Extraction of Phenolic Compounds

##### EVOO Sample

The phenolic fraction was extracted from the Galician EVOO using a liquid–liquid extraction protocol previously reported by Bajoub et al. with some modifications.^18^ Briefly, a portion of 2 (±0.01) g of EVOO was weighed in a conical centrifuge tube (15 mL) and spiked with 25 μL of the IS (methanolic stock solution at a concentration of 500 mg/L) only for the LC–DAD/FLD/MS analysis. After solvent evaporation under N_2_ current, the sample was dissolved in *n*-hexane (1 mL) and extracted three times with 2 mL portions of MeOH/H_2_O (60:40, v/v) by vigorous vortex shaking. All of the supernatants obtained after centrifugation were combined and either directly used for the spectrophotometric assays or evaporated to dryness with a TurboVap Evaporator for LC–DAD/FLD/MS analysis. The remaining residue was redissolved in ACN/H_2_O (50:50, v/v, 1 mL), filtered through a 0.22 μm PVDF syringe filter, and stored at -80 °C until analysis. Before injection into the chromatographic system, an aliquot of the prepared extract was diluted (1:10, v/v) with ACN/H_2_O (50:50, v/v).

##### Clean-Up Procedure of Wp and Bf Phenolic Compounds

An aliquot of the Wp (1 mL) and the total volume of Bf (15 mL) were passed through SPE cartridges according to the method described by Suárez et al. with some modifications.^19^ The retained phenolic compounds were eluted using MeOH (5 mL). Before concentrating the analytes, the spectrophotometric analyses were carried out on an aliquot of this solution. Next, the remaining elution solvent was evaporated to dryness under N_2_ current in a TurboVap evaporator and redissolved in ACN/H_2_O (50:50, v/v, 1 mL). All of the extracts were filtered through 0.22 μm PVDF syringe filters and stored at −80 °C until analysis.

##### Extraction and Clean-Up Procedure of Op Phenolic Compounds

The Op samples were dissolved in *n*-hexane (5 mL) and extracted three times with 5 mL portions of MeOH/H_2_O (60:40, v/v) by vigorous vortex shaking. All supernatants obtained after centrifugation were combined. An aliquot (1 mL) was passed through SPE cartridges, and the retained phenolic compounds were eluted using MeOH (5 mL). In the same way, as for the Wp, one aliquot of this solution was used for the spectrophotometric assays. The remaining elution solvent was evaporated to dryness in a TurboVap evaporator and redissolved in ACN/H_2_O (50:50, v/v, 1 mL). All of the extracts were filtered through 0.22 μm PVDF syringe filters and stored at −80 °C until analysis.

**Figure 1:**
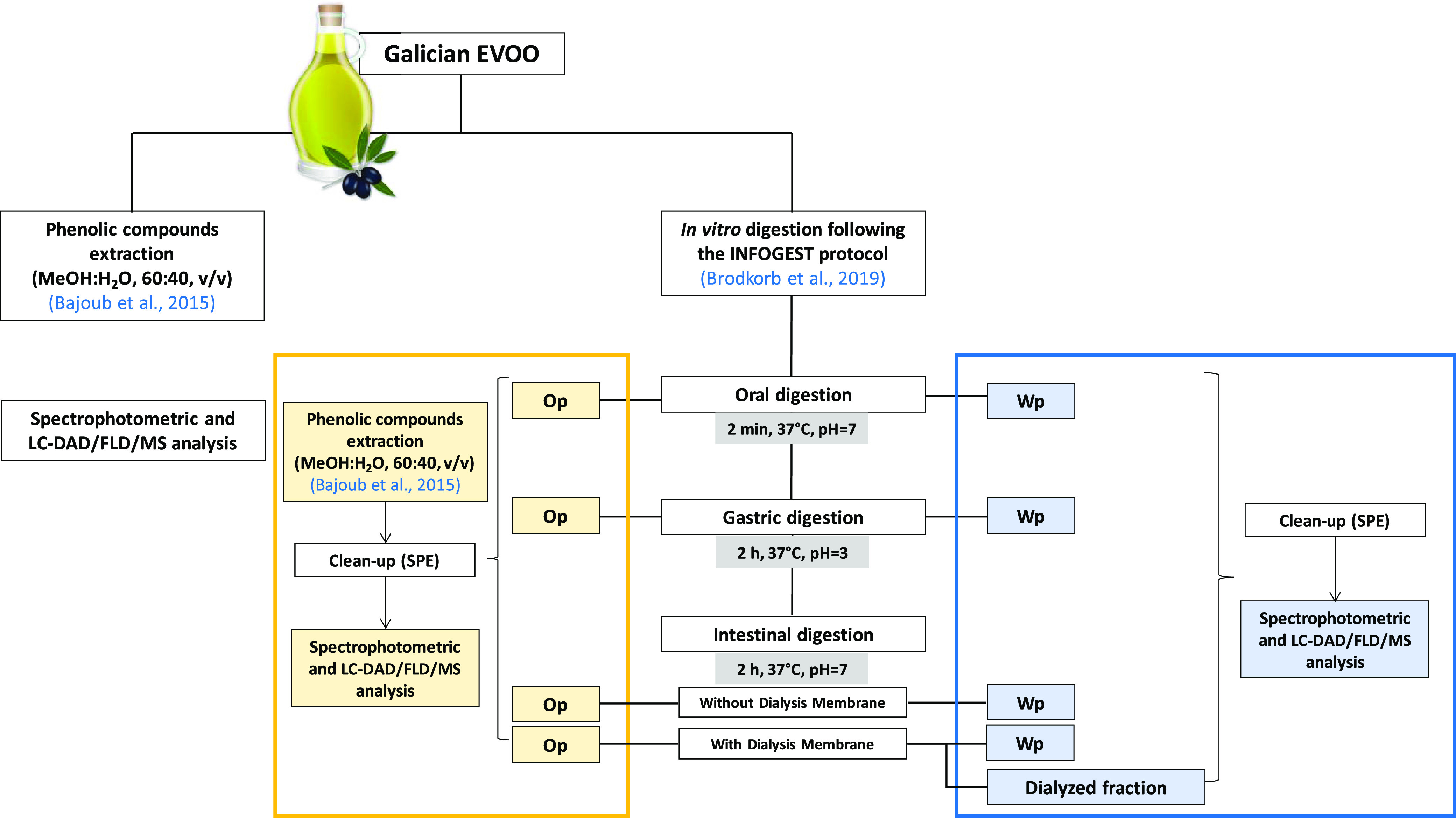
Schematic of the experiments including the fractions collected after each digestion stage and the performed determinations. Nomenclature: oily phase (Op); water phase (Wp).

The schematic of the experimental part, as well as the nomenclature used, is shown in Figure 1.

### Spectrophotometric Analysis of Phenolic Extracts

#### Total Phenolic Compounds (TPC)

TPC was determined using the Folin–Ciocalteu method, as modified by Slinkard and Singleton.^20^ Briefly, the phenolic extract (500 μL) was mixed with the Folin–Ciocalteu reagent (10%, 2.5 mL) and kept for 5 min at room temperature. Then, Na_2_CO_3_ solution (0.7 M, 2 mL) was added to the mix. After 2 h of incubation at room temperature in the dark, a UV–vis spectrophotometer was used to measure the absorbance of the resulting solution at 760 nm. GA was used as standard, and the results were expressed as milligrams of GA equivalents (GAE) per kg of EVOO (mg GAE/kg).

#### *o*-Diphenols

An aliquot of a solution (4 mL) prepared by mixing the phenolic extract (500 μL) and MeOH/H_2_O (50:50, v/v, 4.5 mL) was added to a 5% solution of Na_2_MoO_4_·2H_2_O in EtOH/H_2_O (50:50, v/v, 1 mL) and vortexed for 1 min. After 10 min of incubation at room temperature, the mixture was centrifuged for 5 min at 3000 rpm. *o*-Diphenol compounds were detected at 370 nm and quantified using GA calibration curves. Data were expressed as mg GAE/kg.^5^

#### Antioxidant Capacity (AC)

The AC was assessed by the DPPH method, with some modifications.^21^ The phenolic extract (50 μL) was diluted with a hydroalcoholic solution of ethanol (70%, v/v, 550 μL). The diluted extract was added to a DPPH solution (400 μL). The mixture was vigorously stirred for a few seconds and kept in the dark for 15 min. Absorbance was measured at 517 nm against MeOH. Trolox was used as standard, and the results were expressed as μmoles of Trolox equivalents (TE) per kg of EVOO (μmol TE/kg).

### LC–DAD/FLD/MS Analysis of Phenolic Extracts

An Agilent 1260-LC system (Agilent Technologies, Waldbronn, Germany) was used; it was equipped with a vacuum degassed, a binary pump, an autosampler, a DAD, and a multiple-wavelength FLD. Apart from the two mentioned detectors, the chromatographic system was coupled to a Bruker Daltonics Esquire 2000 ion trap (IT) mass spectrometer (Bruker Daltonik, Bremen, Germany) by means of an electrospray ionization (ESI) source. This platform was chosen because it allowed the simultaneous monitoring of the chromatographic eluent with three different and complementary detectors.

The chromatographic separation was carried out in a Zorbax C_18_ analytical column (4.6 × 150 mm^2^, 1.8 μm particle size) (Agilent Technologies) operating at 25 °C. Analytes of interest were eluted at a flow rate of 0.8 mL/min with acidified H_2_O (0.5% AcH) (phase A) and ACN (phase B) as mobile phases using the following elution gradient: 0–10 min, 5–30% B; 10–12 min, 30–33% B; 12–17 min, 33–38% B; 17–20 min, 38–50% B; 20–23 min, 50–95% B. ACN percentage was finally reduced to the initial conditions (5%) and the column reequilibrated (for about 2 min) before the subsequent injection. An injection volume of 10 μL was set.

The separate compounds were monitored on-line with the FLD, DAD, and ESI-IT MS detectors. In the first one, the excitation and emission wavelengths were set at 280 and 328 nm, respectively. Some other important parameters in that detector were 2.31 Hz for signal acquisition rate, 10 units for photomultiplier (PMT) gain, 5% of zero offset, and 100 luminescence units (LU) of attenuation in analog output. FLD was very useful for the determination of lignans (Pin and Ac-Pin). For the DAD detector, the selected wavelengths were 240, 280, and 330 nm; the latter was the chosen wavelength for the determination of flavonoids (Lut, Api, and Diosmetin (Dios)). Regarding the MS conditions, the IT was operated in full scan mode (*m*/*z* range 50–800) in negative polarity. The ESI parameters were set as follows: capillary voltage, +3200 V; drying gas temperature, 300 °C; drying gas flow, 9 L/min; and nebulizing gas pressure, 30 psi. The skimmers, octopoles, and lenses voltages were tuned considering the average mass, which was set as the target mass value. Additionally, auto MS/MS analyses were carried out to characterize the fragmentation patterns of the compounds under study.

Chromatographic data acquisition was performed using ChemStation B.04.03 software (Agilent Technologies). The mass spectrometer was controlled using the software Esquire Control, and the obtained files were processed with the software Data Analysis 4.0 (Bruker Daltonik).

Another platform, Waters Acquity UPLC H–Class system (Waters, Manchester, U.K.) coupled to a Q-TOF SYNAPT G2 MS (Waters) equipped with an ESI ion source, was used only for qualitative purposes. The ESI-IT MS parameters were transferred to the ESI-QTOF spectrometer.

The identification of the phenolic compounds found in the analyzed samples was based on the use of pure standards (when commercially available), retention time data, high-resolution MS information, and the comparison of the MS/MS spectra with previously published results.^22^ Calibration curves for every pure standard were built using different concentrations of the standard mixture solution and plotting peak areas *vs* concentration levels. When a pure standard was not available, the quantification was made using the calibration curve of a similar (or structurally related) compound: HTy was used for oleuropein aglycone (OlAgl) and related compounds; Ty was used for ligstroside aglycone (LigAgl) and related compounds; lignans were quantified in terms of Pin; Lut was used for Dios; and Ole was used for all elenolic acid (EA) derivatives. The results were expressed in mg/kg of EVOO, as mean ± standard deviation (calculated from four extracts; *n* = 4).

#### Matrix Effect Evaluation

To evaluate the matrix effect on the intestinal fluid (for oral and gastric digestion, matrix effect resulted to be insignificant), the slope of the external calibration curve prepared in ACN/H_2_O (50:50, v/v) and the one from the standard addition of the analyte under study in the Wp collected after intestinal digestion were compared following the equation
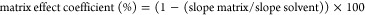
When applying the LC-MS method, negligible matrix effect (matrix effect coefficients lower than 15%) was found for six of the evaluated compounds: HTy (6%), Ty (14%), Van (9%), *p*-Cou (10%), Fer (7%), and Ole (4%). On the other hand, the coefficients obtained for Lut, Api, and Pin showed a response enhancement (greater than 15%) produced by the matrix. To avoid such matrix effect, the determination of these three phenolic compounds (and two related substances) was selectively performed by DAD for Lut, Dios, and Api (at 330 nm) and by FLD for Pin and Ac-Pin (λ_exc_ 280 – λ_em_ 328). Their determination using the chosen detectors and wavelengths was affected by an irrelevant matrix effect.

### *In Vitro* α-Glucosidase Inhibition

EVOO phenolic extracts obtained in the extraction of phenolic compounds and the Bf containing the phenolic compounds able to cross the synthetic membrane were evaporated and redissolved in phosphate buffer before being used in the subsequent *in vitro* inhibitory assay. α-Glucosidase inhibitory activity was assessed by following a previously reported procedure.^23^ Briefly, each reservoir contained PNP-G (2.5 mM), phosphate buffer, and extract or buffer (negative control). The reaction was initiated by adding an enzyme solution (0.28 U/mL, 20 μL). The plates were incubated at 37 °C for 10 min. The rate of release of 4-nitrophenol from PNP-G at 405 nm was measured in an LT-5000 MS ELISA READER from Labtech (Bergamo, Italy) from 0 to 10 min. Acarbose was the positive control.

## Results and Discussion

### Characterization of the Galician EVOO Phenolic Fraction

#### Determination of Individual Phenolic Compounds

A total of 21 phenolic compounds were identified in the selected Galician EVOO (Table 1). They have been grouped within the following six subfamilies: Ole derivatives, ligstroside (Lig) derivatives, simple phenols, phenolic acids, flavonoids, and lignans. EA derivatives (four compounds) that are strictly nonphenolic but structurally related compounds were also determined.

**Table 1:** Phenolic Compounds Determined in the Two Fractions (Op and Wp) (mg/kg of EVOO) after *In Vitro* Digestion (Oral, Gastric, and Intestinal) of Galician EVOO (mg/kg of EVOO)^a^

		oily phase (Op)			water phase (Wp)			
phenolic compounds	EVOO	oral step	gastric step	intestinal step	oral step	gastric step	intestinal step	potential bioaccesibility (%)
oleuropein derivatives								
DOA	301.40 ± 13.04	113.58 ± 14.66	5.49 ± 0.23	n.d.	172.97 ± 6.84	5.91 ± 0.57	n.d.	
OlAgl (Is I)	1.25 ± 0.11	8.38 ± 1.27	0.82 ± 0.28	n.d.	8.26 ± 0.68	n.d.	n.d.	
OlAgl (main peak)	80.33 ± 2.82	80.88 ± 6.56	52.07 ± 3.33	2.16 ± 0.80	22.59 ± 1.54	38.64 ± 4.81	0.68 ± 0.72	0.8
OlAgl (Is II)	13.40 ± 1.83	13.60 ± 1.46	5.19 ± 0.26	n.d.	5.36 ± 0.35	0.64 ± 0.04	n.d.	
	396.38	216.44	63.57	2.16	209.18	45.19	0.68	
ligstroside derivatives								
DLA	515.11 ± 17.02	289.80 ± 45.92	37.65 ± 4.74	n.d.	112.45 ± 8.34	1.86 ± 0.28	n.d.	
LigAgl (Is I)	16.41 ± 0.35	39.71 ± 2.03	11.18 ± 1.09	n.d.	15.23 ± 1.77	n.d.	n.d.	
LigAgl (Main peak)	234.08 ± 22.30	112.90 ± 0.80	47.60 ± 4.38	5.64 ± 0.70	15.54 ± 1.56	8.01 ± 0.88	n.d.	
LigAgl (Is IV)	51.73 ± 6.22	48.02 ± 1.72	19.43 ± 1.47	n.d.	12.40 ± 0.87	n.d.	n.d.	
	817.33	490.43	115.86	5.64	155.62	9.87		
simple phenols								
O-HTy	0.05 ± 0.01	n.d.	n.d.	n.d.	0.07 ± 0.01	n.d.	n.d.	
HTy	6.22 ± 0.23	0.45 ± 0.02	0.61 ± 0.03	0.047 ± 0.001	12.44 ± 0.65	8.38 ± 0.48	18.79 ± 0.38	302
Ty	4.95 ± 0.21	0.48 ± 0.05	0.57 ± 0.20	n.d.	6.43 ± 0.26	3.82 ± 0.47	8.51 ± 1.07	172
HTy-Ac	0.29 ± 0.02	0.05 ± 0.03	0.16 ± 0.08	1.95 ± 0.29	0.67 ± 0.04	6.87 ± 0.86	12.14 ± 1.06	4186
	11.51	0.98	1.34	2.00	19.61	19.07	39.44	
phenolic acids								
GA	0.51 ± 0.04	0.02 ± 0.01	n.d.	n.d.	0.59 ± 0.08	0.24 ± 0.02	n.d.	
Van	0.21 ± 0.02	n.d.	0.08 ± 0.01	n.d.	0.21 ± 0.03	0.07 ± 0.01	n.d.	
*p*-Cou	0.19 ± 0.01	n.d.	0.04 ± 0.01	n.d.	0.15 ± 0.01	0.028 ± 0.002	n.d.	
	0.91	0.02	0.12		0.95	0.34		
flavonoids								
Lut^b^	3.80 ± 0.43	2.03 ± 0.15	2.24 ± 0.11	0.85 ± 0.10	1.06 ± 0.12	0.54 ± 0.04	2.04 ± 0.20	54
Api^b^	0.79 ± 0.08	0.60 ± 0.05	0.57 ± 0.04	0.39 ± 0.06	0.036 ± 0.004	0.022 ± 0.002	0.18 ± 0.01	23
Dios^b^	0.38 ± 0.05	0.33 ± 0.02	0.31 ± 0.02	0.21 ± 0.03	0.02 ± 0.01	0.002 ± 0.001	0.10 ± 0.02	26
	4.97	2.96	3.12	1.45	1.12	0.56	2.32	
lignans								
Syr	0.043 ± 0.004	0.09 ± 0.01	0.072 ± 0.004	0.025 ± 0.003	0.05 ± 0.01	0.09 ± 0.01	n.d.	
Pin^c^	1.81 ± 0.10	1.64 ± 0.09	1.20 ± 0.06	0.80 ± 0.08	0.173 ± 0.004	0.29 ± 0.04	0.46 ± 0.06	25
Ac-Pin^c^	0.17 ± 0.01	0.13 ± 0.01	0.09 ± 0.01	0.056 ± 0.005	0.036 ± 0.002	0.06 ± 0.01	0.10 ± 0.01	59
	2.02	1.86	1.36	0.88	0.26	0.44	0.56	
total phenolic compounds	1233.12	712.69	185.37	12.13	386.74	75.47	43.00	
Nonphenolic But Structurally Related Compounds								
elenolic acid derivatives								
DEA	3.54 ± 0.46	0.10 ± 0.04	n.d.	n.d.	3.82 ± 0.42	2.21 ± 0.32	0.71 ± 0.09	20
Desoxy-EA	113.76 ± 14.42	21.42 ± 2.68	8.75 ± 1.19	0.46 ± 0.09	50.82 ± 3.74	28.65 ± 2.58	6.88 ± 1.17	6.0
Hy-EA	0.43 ± 0.07	n.d.	n.d.	n.d.	0.37 ± 0.06	0.14 ± 0.09	0.29 ± 0.06	67
EA	356.21 ± 27.12	11.36 ± 1.24	8.877 ± 0.002	n.d.	274.04 ± 35.27	12.17 ± 0.30	n.d.	
	473.94	32.88	17.63	0.46	329.05	43.17	7.88	

a Abbreviations: DOA: dialdehydic form of decarboxymethyl oleuropein aglycone or dialdehydic form of decarboxymethyl elenolic acid linked to hydroxytyrosol or oleacein; OlAgl (main peak): oleuropein aglycone (main peak); OlAgl (Is I): oleuropein aglycone (isomer I); OlAgl (Is II): oleuropein aglycone (isomer II); DLA: dialdehydic form of decarboxymethyl ligstroside aglycone or dialdehydic form of decarboxymethyl elenolic acid linked to tyrosol or oleocanthal; LigAgl (main peak): ligstroside aglycone (main peak); LigAgl (Is I): ligstroside aglycone (isomer I); LigAgl (Is IV): ligstroside aglycone (isomer IV); O-HTy: oxidized hydroxytyrosol; HTy: hydroxytyrosol or 3,4-dihydroxyphenylethanol; Ty: tyrosol or *p*-hydroxyphenylethanol; HTy-Ac: hydroxytyrosol acetate; Van: vanillic acid; *p*-Cou: *p*-coumaric acid; Lut: luteolin; Api: apigenin; Dios: diosmetin; Syr: syringaresinol; Pin: pinoresinol; Ac-Pin: acetoxypinoresinol; DEA: decarboxymethylated form of elenolic acid or dialdehydic form of decarboxymethyl of elenolic acid; Desoxy-EA: Desoxy elenolic acid; Hy-EA: hydroxy elenolic acid or hydroxylated form of elenolic acid; EA: elenolic acid.

b Lut, Api, and Dios were determined by DAD (λ = 330 nm).

c Pin and Ac-Pin were determined by FLD (280–328).

Secoiridoids, which are complex phenols including Ole and Lig derivatives, are the most abundant family in the EVOO polar fraction. These compounds represented 98% of the total phenolics in the EVOO under study, where Ole derivatives accounted for 32% and Lig derivatives for the remaining 66%. This finding was in agreement with the percentages found in other Spanish widely known varieties, where Lig derivatives were the most abundant too, accounting for 79% (Cornicabra EVOO) and 65% (Picual EVOO).^24^ DOA (also known as oleacein), followed by OlAgl (main peak), and its isomers (isomers II and I) were the most remarkable Ole derivatives with the following concentrations: 301.40, 80.33, 13.40, and 1.25 mg/kg, respectively. DLA (also known as oleocanthal) and LigAgl (main peak) showed the highest concentration (515.11 and 234.08 mg/kg, respectively) within the Lig derivatives group. The content of these derivatives together with those of HTy and Ty have been correlated in humans with increased contents of antioxidant LDL and nutrigenomic effects,^25^ hence their importance.

HTy and Ty were the most relevant simple phenols in VOO. Their concentrations in the selected EVOO were 6.22 and 4.95 mg/kg for HTy and Ty, respectively. Compared with Picual (HTy: 9.7 mg/kg and Ty: 5.6 mg/kg) and Cornicabra (HTy: 0.96 mg/kg and Ty: 2.1 mg/kg) EVOOs,^24^ the Galician EVOO under study revealed similar concentrations to Picual EVOO and a higher amount of both simple phenols in relation to Cornicabra EVOO. It is well known that HTy is one of the most important antioxidants in VOO.^26^ In fact, a wide variety of HTy biological properties have been associated with its strong antioxidant activity. Ty has also been shown to be an effective cellular antioxidant.^27^ Other simple phenols, namely, oxidized hydroxytyrosol (O-HTy) and HTy-Ac, were found at low concentrations.

Several phenolic acids such as caffeic, cinnamic, Fer, GA, *p-* and *o-*Cou, *p-*hydroxybenzoic, protocatechuic, syringic, and Van have been detected in VOO at low concentrations (quantities <1 mg/kg). As can be seen in Table 1, only GA, Van, and *p*-Cou were quantified in the Galician EVOO under study. Even though they are present in relatively small amounts, some authors pointed out that these compounds could be potential markers of the geographical origin of the olive variety.^4^

Three flavonoids were found in the target EVOO: Lut, Api, and Dios at the following concentrations 3.80, 0.79, and 0.38 mg/kg, respectively. The beneficial effects of this family of compounds have been attributed to their antioxidant activity and influence on cell redox state.^28^

Lignans typically found in VOOs include Pin, syringaresinol (Syr), and Ac-Pin. According to Brenes and co-workers, their concentrations range from 11.7 to 41.2 mg/kg for Pin and from 2.7 to 66.9 mg/kg for Ac-Pin,^29^ although it is evident that it depends on a number of factors. The studied EVOO presented low amounts of lignans, Pin being found at the highest concentration (1.81 mg/kg). The biological relevance of these compounds lies in the fact that they have been associated with protection against LDL oxidation and inhibition of cancer cells growth.^30^

Nonphenolic but structurally related compounds include, as previously stated, EA and its derivatives: dialdehydic form of decarboxymethyl EA (DEA), Desoxy-EA, and hydroxy EA (Hy-EA). They are generated as a result of the hydrolysis of secoiridoids during the VOO elaboration process. EA was the most abundant in the selected EVOO (356.21 mg/kg).

#### Spectrophotometric Assays

**Figure 2:**
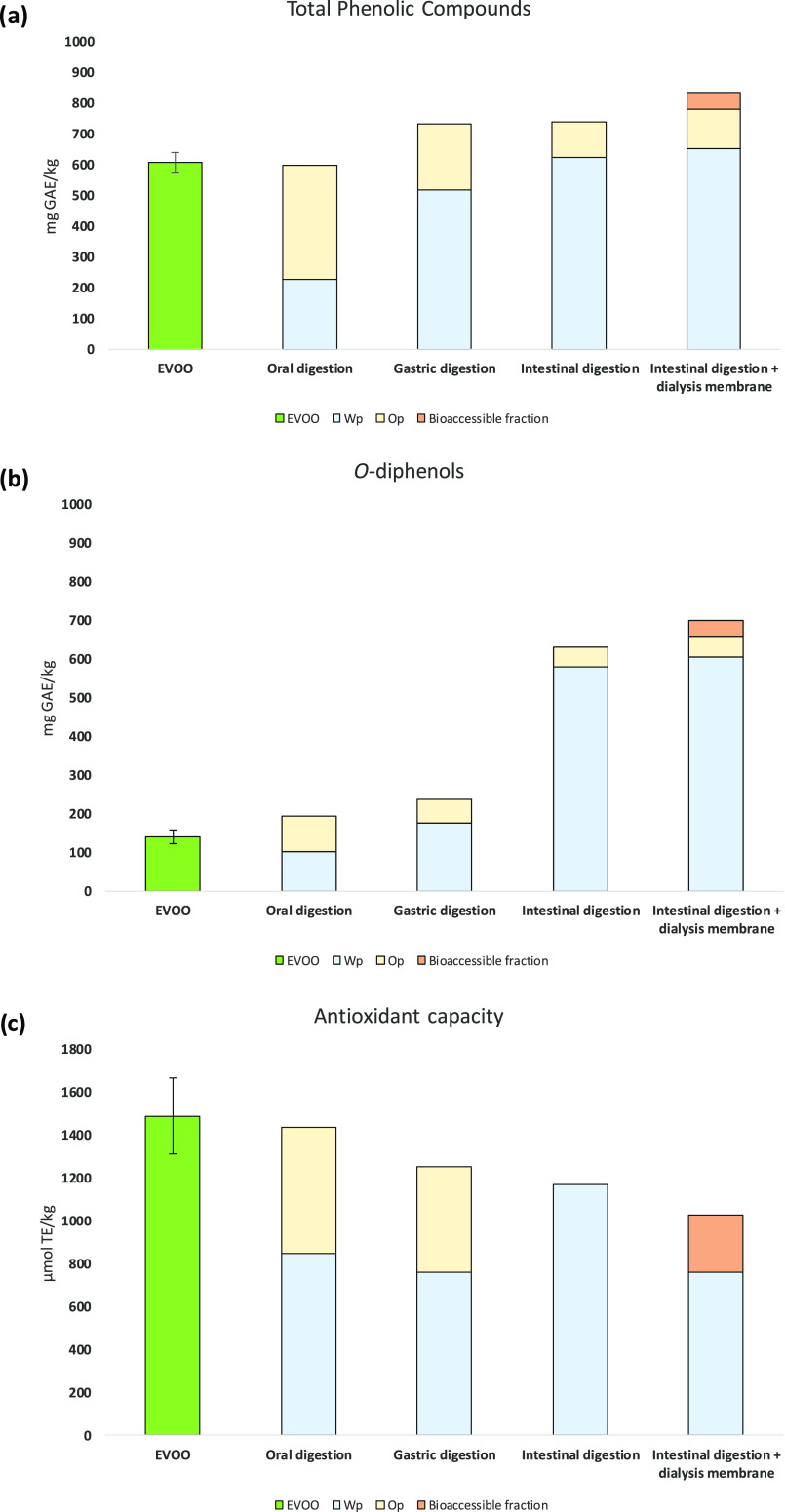
Total phenolic compounds (TPC), *o*-diphenols, and antioxidant capacity (AC) of Galician EVOO before *in vitro* digestion (MeOH extract) and after *in vitro* digestion (oral digestion, gastric digestion, intestinal digestion, and intestinal digestion + dialysis membrane). GAE: gallic acid equivalents; TE: Trolox equivalents. It has not been possible to determine the AC in the oily phase of the intestinal digestion and the intestinal digestion + dialysis membrane.

The TPC was determined by the Folin–Ciocalteu colorimetric method, which is a simple, repeatable, and robust procedure that generates a global value of the phenolic content. Figure 2 shows the TPC for the studied Galician EVOO (607.9 mg GAE/kg), which can be considered an outstanding value compared with published data; only EVOOs from Picual variety, which are well known for their high phenolic content, have shown even higher results (∼1000 mg GAE/kg).^31^ The concentration of *o*-diphenols represents 23% of the TPC (139.5 mg GAE/kg), which is in agreement with our previous results where the percentage of *o*-diphenols represented 25% of the TPC for EVOOs elaborated by co-crushing “Brava Gallega” and “Mansa de Figueiredo”.^5^ It must be noticed that these percentages apply just to the results obtained by spectrophotometric methods; the sum of individually quantified phenolic compounds has been proven to be in disagreement with nonspecific global results, as widely discussed by Olmo-García et al.^32^

Antioxidant properties have been traditionally studied in chemical extracts by applying assays to evaluate radical scavenging capacity (DPPH and ABTS) and FRAP.^31,33^ In the present study, the AC was determined by the DPPH method. As can be seen in Figure 2, phenolic extract from the target EVOO showed high AC (1490.8 μmol TE/kg). Negro et al. reported that AC is a good index for representing oil quality because it is positively correlated to phenolic compounds concentration.^34^ It is well known that the antioxidant activity is higher for DOA and OlAgl (oleuropein derivatives), HTy (simple phenol), GA (phenolic acid), and Lut (flavonoid) because of the presence in their chemical structure of a hydroxyl group in the ortho position;^35^ DOA which bears a dialdehydic form of EA linked to HTy exhibits an AC higher than that of OlAgl.^36^

### Transformation of Phenolic Compounds after *In Vitro* Digestion

The *in vitro* digestion model involves three steps (oral, gastric, and intestinal digestion). The study design was performed to be able to evaluate the effect of each step of the digestion on VOO phenolics and, therefore, their potential bioaccessibility and bioaccessibility. This set of experiments made it possible to delve into the transformations that the phenolic compounds suffer throughout the gastrointestinal tract.

### Oral Digestion

At the end of the oral digestion, two fractions were obtained: Wp and Op. The determination of the phenolic compounds and AC in both fractions was carried out by applying the procedures described in the Extraction of Phenolic Compounds Section. The obtained results are shown in Table 1 and Figure 2.

During oral digestion, the pH was not modified, and no enzymes were added, which could alter the chemical structure of the analytes under study. Therefore, the distribution of phenolic compounds between the Wp and Op was clearly regulated by their polarity.^14,37^ As seen in Table 1, the most polar phenolic compounds (*i.e.*, phenolic acids, and simple phenols) were mainly detected in the Wp; meanwhile, the most hydrophobic compounds (lignans and flavonoids) were predominantly present in the Op, except for Lut. EA derivatives were mostly found in the Wp.

Secoiridoids showed high stability during the oral digestion and unlike other chemical compounds, they were distributed between both fractions. Among secoiridoids, DOA was mainly present in the Wp (60%); meanwhile, DLA was mainly present in the Op (72%) (Table 1). Notwithstanding, EVOO native DOA, DLA, and LigAgl (main peak) were hydrolyzed (5, 22, and 45%, respectively) leading to an increase in both HTy and Ty. EA derivatives were reduced by around 24% with respect to the EVOO native amount and were primarily detected in the Wp (91%). These findings contrast with those from Quintero-Flórez et al., who also quantified the individual phenolic compounds found in the Wp and the Op after oral, gastric, and intestinal digestion of EVOOs obtained from Picual, Blanqueta, Sevillana, Habichuelero, and Chetoui varieties.^14^ Surprisingly, they observed that secoiridoids were hydrolyzed to a high extent (80–84%) during oral digestion with a consequent increase of HTy, Ty, and Ty hexoside.

The results obtained by spectrophotometric methods were consistent with those obtained by LC–DAD/FLD/MS (Figure 2). The sum of TPC in the Wp (225.5 mg GAE/kg) and Op (373.1 mg GAE/kg) gave a total value of 598.6 mg GAE/kg, which was very similar to the TPC measured in the selected EVOO (607.9 mg GAE/kg). TPC in Wp accounted for 38% and Op accounted for 62%. The same behavior was observed for *o*-diphenols, whose total content in the Wp (100.9 mg GAE/kg) and Op (93.1 mg GAE/kg) gave 194 mg GAE/kg, a slightly higher value than that initially obtained for the EVOO (139.5 mg GAE/kg). Presumably, this was a consequence of the increase in HTy concentration (Wp: 52% and Op: 48%). Finally, the sum of the AC of the Wp (850.3 μmol Trolox/kg) and the Op (587.8 μmol Trolox/kg) was of the same order of magnitude as that obtained for the Galician EVOO (1490.8 μmol Trolox/kg) (Wp: 59% and Op: 41%, identical percentages to those observed for *o*-diphenols). It should be noted that it has not been possible to compare the spectrophotometric results with those obtained by other authors since there are no available literature reports where both fractions have been analyzed after oral digestion.

### Gastric Digestion

At the end of the gastric digestion, both fractions (Wp and Op) were subjected to the determinations described in the Extraction of Phenolic Compounds Section. The obtained results are presented in Table 1 and Figure 2.

In contrast to oral digestion, the gastric digestion conditions affected the stability of the studied compounds since it takes place at acid pH and involves the addition of pepsin and lipase enzymes (Table 1). Although the main phenolic compounds in both fractions corresponded again to secoiridoid derivatives, they were unstable under gastric conditions, and their content drastically diminished with respect to the oral digestion step: the reduction for Ole derivatives was around 75%, and that for Lig derivatives was around 80%. In this study, extensive hydrolysis of the secoiridoids (mainly DOA and DLA) was registered. Previous studies performed by other authors pointed out a high increase of simple phenols (free HTy and Ty),^14,38^ in contrast to the slight hydrolysis observed by Romero et al. and Soler et al.^37,39^ EA derivatives content decreased by around 83% with respect to the oral digestion and they were mainly detected in the Wp (43.17 *vs* 17.63 mg/kg).

Ty has been described as a degradation product of LigAgl^40^ and HTy can be released from its precursors (Ole secoiridoids and HTy-Ac) during digestion.^9^ Moreover, Pereira-Caro et al. showed that HTy-Ac presented slight hydrolysis to free HTy after gastric digestion exclusively due to the influence of acid conditions.^13^ In the present work, the concentration of HTy-Ac increased 10-fold with respect to the oral digestion; meanwhile, the concentrations of HTy decreased 1.5-fold. This trend could indicate that Ole derivatives, which are unstable at gastric conditions, were hydrolyzed to both HTy-Ac and HTy. Similar results have been described by Quintero-Flórez et al. where increased concentrations of HTy-Ac and decreased concentrations of HTy were observed after gastric digestion of the studied EVOOs.^14^

Flavonoids and lignans were quite stable to gastric conditions and were mainly recovered in the Op.^14,37^ Lut, the main flavonoid, and Pin, the main lignan, showed high stability after gastric digestion.

The chemical transformation of phenolic compounds as a consequence of gastric digestion caused an increase in Wp TPC and *o*-diphenols content (Figure 2). TPC in the Wp was practically doubled with respect to the previous stage, from 225.2 to 519.1 mg GAE/kg. However, this behavior was not reflected by the individual quantification of phenolic compounds. According to the information provided in Table 1, secoridoid derivatives drastically reduced their content with gastric conditions in both fractions. This fact could demonstrate that secoiridoids were not properly determined or “captured” by the spectrophotometric assay which, in turn, seemed to be more trustworthy and suitable to determine simple structures such as HTy and HTy-Ac; indeed, the last one showed a large increase after hydrolysis. On the other hand, the TPC in the Op was reduced by around 42%, from 373.1 to 215.2 mg GAE/kg (Figure 2).

Concerning the *o*-diphenols, the content in the Wp was almost twice higher after gastric digestion than after oral digestion (Figure 2b). This increase from 100.9 up to 175.0 mg GAE/kg may be due to the HTy-Ac increase. The content of *o*-diphenols in the Op slightly decreased from 93.1 to 62.2 mg GAE/kg, suggesting that these compounds were barely altered either by the pH or by the activity of the enzymes.

Despite the fact that the TPC measured by the Folin–Ciocalteu assay in the gastric phase was twice the phenolic content in the oral phase, the AC suffered a mild decrease (Figure 2c). Considering that the total concentration of simple phenols remains almost constant (Table 1), the slight decrease observed in Wp could be due to the reduction in GA and Lut, Api, and Dios concentrations. In the same way as for oral digestion, there are no available reports where both fractions have been analyzed after the gastric digestion.

### Intestinal Digestion

At the end of the intestinal digestion, phenolic compounds and the AC were determined in both fractions (Wp and Op) as described in the Extraction of Phenolic Compounds Section. The obtained results are gathered in Table 1 and Figure 2.

The Wp represents the potential bioaccessible fraction which is the fraction that is released from the EVOO matrix in the gastrointestinal tract and becomes available for absorption.^41^ The potential bioaccessible fraction contains phenolic substances soluble in the Wp, as well as phenolic compounds that could be present in the oil core of the micelles emulsified by bile salts, depending on their lipophilic nature.^8^ Simple phenols (HTy, Ty, and HTy-Ac) and flavonoids (Lut) were mainly recovered in the Wp; meanwhile, lignans (Pin y Ac-Pin) were quite stable to duodenal digestion conditions and remained in the Op (Table 1). The stability of secoiridoids and structurally related compounds was very low when they were exposed to small-intestinal conditions. EA derivatives almost disappeared and the hydrolysis of secoiridoids was again responsible for the release of simple phenols HTy, HTy-Ac, and Ty,^9,37,42^ which were stable under the conditions of intestinal digestion.^13^ As in the present study, Quintero-Flórez et al. described an increase in simple phenols concentration related to the decrease in the secoiridoids content; the remaining secoiridoid derivatives were mainly found in the Wp.^14^ The reduction of the phenolic compounds in the Op could be linked to their migration to the micelles formed by the bile salts.

Table 1 also shows the potential bioaccessibility values for the phenolic and nonphenolic but structurally related compounds after intestinal digestion of the Galician EVOO calculated by eq 1
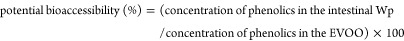
1Simple phenols had the highest potential bioaccessibility values, followed by flavonoids, lignans, EA derivatives, and the OlAgl (main peak). Among simple phenols, HTy-Ac is the most potential bioaccessible (4186%), followed by HTy (302%) and Ty (172%). An HTy bioaccessibility of 132.2% has been calculated by Rubió et al. after *in vitro* digestion of an olive oil extract.^9^ Quintero-Flórez et al. reported a bioaccessible index ranging from 212 to 2452% for HTy and from 76 to 163% for Ty depending on the cultivar.^14^ The bioaccessibility of HTy-Ac was over 100% in all varieties except for the “Picual” VOO (17%).^14^ With respect to flavonoids, the potential bioaccessibility values found in the current study for Lut, Dios, and Api were 54, 26, and 23%, respectively. These results contrast with the findings from Quintero-Flórez et al., where Lut and Api were not bioaccessible or had very low bioaccessibility values.^14^ Lut was also found in the bioaccessible fraction after *in vitro* digestion of an olive extract at very low concentrations (bioaccessibility percentage of 14.6%) by Rubió et al.^9^ Among lignans, the potential bioaccessibility values of Ac-Pin and Pin were 59 and 25%, respectively. In general, Ac-Pin tended to be more bioaccessible than Pin in all of the EVOOs evaluated by Quintero-Flórez et al.^14^ EA derivatives and OlAgl (main peak) showed low potential bioaccessibility.

The phenolic compounds that remain in the Op, mainly lignans and flavonoids, could interact with the intestinal microbiota, which facilitates their degradation and transformation into substances with low molecular weight and potentially absorbable structures in the colon.^43,44^ In fact, lignans could be metabolized to enterodiol and enterolactone while flavonoids could be hydrolyzed to simple phenolic acids.^45,46^ In addition, all of those secoiridoid derivatives not finally absorbed in the small intestine could reach the colon, acquiring prebiotic properties if bacterial groups such as *Bifidobacteria* and *Lactobacillus* are able to use them as a carbon source.^38^

Figure 2 shows how the Wp TPC slightly increased to 624.7 mg GAE/kg (increment of 17% compared to the previous stage). The Op presented a non-negligible TPC (114.0 mg GAE/kg), which represented a percentage of 15% of the TPC after intestinal digestion. The hydrolysis of secoiridoids to HTy and HTy-Ac was probably responsible for the increase of *o*-diphenols concentration until 579.7 mg GAE/kg in the Wp. The content of *o*-diphenols in the Op was reduced with respect to gastric digestion up to 52.3 mg/kg (representing 37% of the content in the EVOO and 8% with respect to the total content in the intestinal phase). The increase in the Wp AC (35%) was of the same order as that of the Wp TPC (17%) but it did not correspond to the trend observed for the *o*-diphenols (70% increase). On the other hand, the Op does not show AC. Several studies have evaluated the bioaccessibility values using spectrophotometric methods^8,10−12,47^ supported that *in vitro* digestion is a crucial step that releases a high amount of phenolics with low molecular structures and antioxidant compounds.

### Estimation of Bioaccesibility

A dialysis membrane was incorporated during the intestinal digestion with the aim of providing more accurate bioaccessibility estimations at the large intestine level. At the end of the intestinal digestion, three fractions were obtained: the dialyzed fraction (containing those compounds which were able to cross the dialysis membrane and representing the bioaccesibility), Wp, and Op at the intestinal level (both together representing the fraction directed to colon). The unabsorbed phenolic compounds, which remain in the intestinal digested fraction, are transported to the colon. Once there, they could be absorbed by the epithelium as native compounds, released, and metabolized by colonic bacteria before being absorbed, or excreted without further metabolism.^48,49^

The percent of bioaccessibility and fraction directed to colon were estimated as follows:

(1) The bioaccessibility (%) was calculated using eq 2 as the dialyzable fraction of phenolic compounds in relation to the content of phenolic compounds of the raw material.
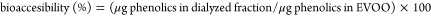
2
(2) The fraction directed to colon (%) was calculated using eq 3 as the content of phenolic compounds in the intestinal digest (Wp + Op) in relation to the content of phenolic compounds of the raw material.
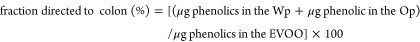
3

**Figure 3:**
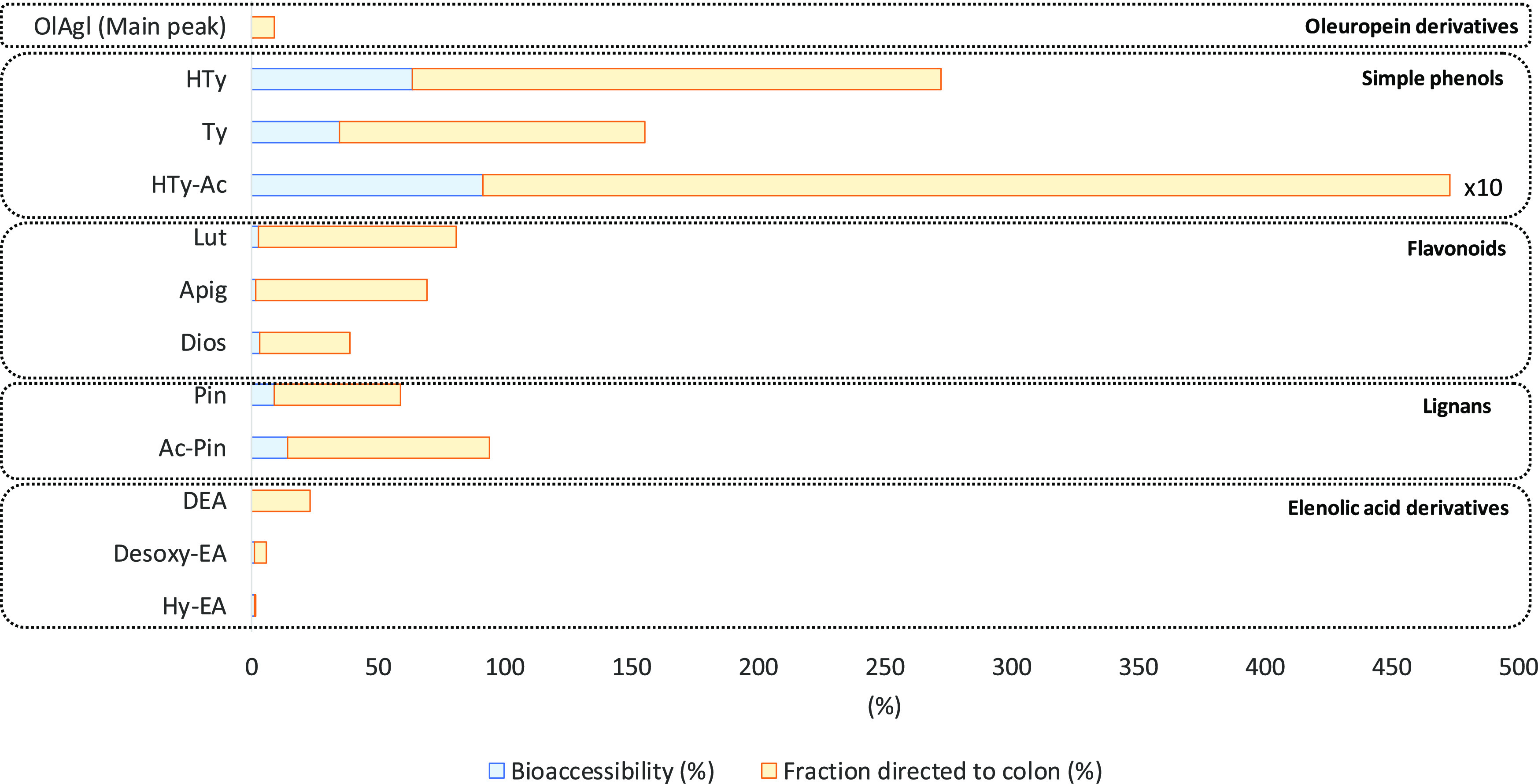
Bioaccessibility (%) and fraction directed to colon (%) of oleuropein derivatives, simple phenols, flavonoids, lignans, and elenolic acid derivatives in the Galician EVOO after *in vitro* digestion as determined by LC–DAD/FLD/MS. OlAgl (main peak): oleuropein aglycone (main peak); HTy: hydroxytyrosol or 3,4-dihydroxyphenylethanol; Ty: tyrosol or *p*-hydroxyphenylethanol; HTy-Ac: hydroxytyrosol acetate; Lut: luteolin; Api: apigenin; Dios: diosmetin; Pin: pinoresinol; Ac-Pin: acetoxypinoresinol; DEA: decarboxymethylated form of elenolic acid or dialdehydic form of decarboxymethyl of elenolic acid; Desoxy-EA: Desoxy elenolic acid; Hy-EA: hydroxy elenolic acid or hydroxylated form of elenolic acid.

Table 2 and Figure 3 show the bioaccessibility and fraction directed to colon of nine phenolic compounds belonging to secoiridoid, simple phenol, flavonoid, and lignan families, and three EA derivatives present in the fractions obtained at the end of the intestinal digestion (dialyzed fraction, Wp and Op). Table 2 shows the amount (μg) of the compounds in each fraction, while Figure 3 shows the percentage of the compounds in each fraction.

**Table 2:** Phenolic Compounds (μg Phenolic Compound in Each Fraction) Determined in the Dialyzed Fraction (Bioaccessibility) and in the Wp and Op (Fraction Directed to Colon) after *In Vitro* Digestion of Galician EVOO as Measured by LC–DAD/FLD/MS^a^

		bioaccessibility		fractions to colon		
phenolic compounds	EVOO	dialyzed fraction (μg)	%	Wp (μg)	Op (μg)	%
oleuropein derivatives						
OlAgl (main peak)	401.65 ± 14.10	1.22 ± 0.13	0.3	13.94 ± 0.67	21.03 ± 2.55	8.7
simple phenols						
HTy	31.10 ± 1.14	19.83 ± 0.98	64	64.78 ± 5.80	0.006 ± 0.005	208
Ty	24.77 ± 1.05	8.61 ± 0.54	35	29.87 ± 1.09	n.d.	121
HTy-Ac	1.47 ± 0.12	13.46 ± 0.42	916	50.13 ± 5.28	5.95 ± 0.81	3815
flavonoids						
Lut^b^	19.02 ± 2.15	0.54 ± 0.04	2.8	12.41 ± 2.29	2.39 ± 0.37	78
Api^b^	3.94 ± 0.39	0.06 ± 0.01	1.5	1.29 ± 0.04	1.38 ± 0.17	68
Dios^b^	1.90 ± 0.27	0.06 ± 0.01	3.2	0.13 ± 0.07	0.55 ± 0.06	36
lignans						
Pin^c^	9.03 ± 0.49	0.81 ± 0.09	9.0	2.10 ± 0.07	2.39 ± 0.31	50
Ac-Pin^c^	0.85 ± 0.03	0.12 ± 0.02	14	0.46 ± 0.01	0.22 ± 0.03	80
Nonphenolic But Structurally Related Compounds						
elenolic acid derivatives						
DEA	17.68 ± 2.30	n.d.		4.12 ± 0.21	n.d.	23
Desoxy-EA	568.81 ± 72.12	5.52 ± 0.87	1.0	27.67 ± 2.21	1.12 ± 0.16	5.1
Hy-EA	2.17 ± 0.34	0.02 ± 0.01	1.0	0.02 ± 0.01	n.d.	0.9

a Abbreviations: OlAgl (main peak): oleuropein aglycone (main peak); HTy: hydroxytyrosol or 3,4-dihydroxyphenylethanol; Ty: tyrosol or *p*-hydroxyphenylethanol; HTy-Ac: hydroxytyrosol acetate; Lut: luteolin; Api: apigenin; Dios: diosmetin; Pin: pinoresinol; Ac-Pin: acetoxypinoresinol; DEA: decarboxymethylated form of elenolic acid or dialdehydic form of decarboxymethyl of elenolic acid; Desoxy-EA: Desoxy elenolic acid; Hy-EA: Hydroxy elenolic acid or hydroxylated form of elenolic acid.

b Lut, Api, and Dios were determined by DAD (λ = 330 nm).

c Pin and Ac-Pin were determined by FLD (280–328).

As seen in Table 2, simple phenols were the main phenolic compounds in the dialyzed fraction (HTy, HTy-Ac, and Ty amounts were 19.86, 13.46, and 8.61 μg, respectively) followed by Desoxy-EA (5.52 μg), OlAgl (main peak) (1.22 μg), Pin (0.81 μg), and Lut (0.54 μg). The compositions of the Wp were 64.78, 50.13, and 29.87 μg for HTy, HTy-Ac, and Ty, respectively, followed by Desoxy-EA (27.67 μg), OlAgl (main peak) (13.94 μg), Lut (12.41 μg), and Pin (2.10 μg). A non-negligible content of phenolics was detected in the Op, where the hydrophobic compounds (lignans and flavonoids) were the predominant ones (Pin and Lut values were 2.39 μg each) together with HTy-Ac (5.95 μg) and OlAgl (21.03 μg). Such results were partially expected due to the relative lipophilic nature of lignans and flavonoids according to the discussion carried out throughout the manuscript.

Figure 3 depicts the bioaccessibility and fraction directed to colon percentages. The most bioaccessible compounds were the simple phenols (HTy-Ac, HTy, and Ty, with percentages of 916, 64, and 35%, respectively), also showing the highest fraction to colon (3815, 209, and 121% for HTy-Ac, HTy, and Ty, respectively). Among flavonoids, Dios and Lut showed similar bioaccessibility (3.0%); however, Lut stood out for its fraction directed to colon (78%). Both lignans (Ac-Pin and Pin) were more bioaccessible than flavonoids (14 and 9.1%, respectively) and also stood out for their fraction directed to colon (80 and 50%, respectively). It should be noted that phenolic acids and Lig derivatives were unstable in the intestinal conditions and disappeared. EA derivatives showed low bioaccessibility (Desoxy-EA: 1.0% and Hy-EA: 1.0%), and their fraction directed to colon was higher (DEA: 23%, Desoxy-EA: 5.1% and Hy-EA: 0.9%).

These three fractions (dialyzed fraction, Wp, and Op) at the intestinal level were also analyzed spectrophotometrically (Figure 2). Although the results obtained for the TPC and *o*-diphenols in the Wp and the Op were quite similar to those obtained without the dialysis membrane and discussed above, the TPC and the *o*-diphenols in the dialyzed fraction (*i.e.*, the bioaccessible fraction) represented 6.7 and 7.5% of the total quantities, respectively. With respect to the AC, the bioaccessible fraction accounted for 13% of the total capacity. As previously observed without dialysis membrane, the Op did not show AC; the Wp decreased because part of the compounds was able to cross the membrane.

Dinnella et al., Seiquer et al., and Borges et al. assessed the bioavailability after the *in vitro* digestion when the digested extracts (considered as the bioaccessible fraction) were subjected to a bioavailability study with Caco-2 cells.^8,10,11^ Dinnella et al. found that absorption of TPC ranged from 17 to 35% for several Oliarola del Bradano EVOOs, from 17 to 18% for Maiatica EVOOs, and from 16 to 36% for Coratina EVOOs.^8^ Seiquer et al. reported that only 25% of the TPC from digested Picual EVOOs crossed the intestinal cells.^10^ Results obtained for Arbequina EVOOs from Brazil and Spain showed that absorption of TPC ranged from 32.5 to 110%.^11^ The AC assessed in the bioavailability fraction of Picual EVOOs was 2% from the initial solution (0.65 mmol Trolox/kg); meanwhile, the AC in Arbequina EVOOs led to 30–52% of DPPH activity.^10,11^

### Antidiabetic Potential of the Bioaccessible Fraction

The inhibitors of α-glucosidase can modulate the absorption of glucose leading to delayed glycemic response and reduced postprandial hyperglycemia.^50^ The ability of the phenolic-rich extracts from EVOOs to inhibit α-glucosidase activity has been reported previously.^7,51,52^ However, to date, no inhibitory effects on α-glucosidase activity for any EVOO considering *in vitro* digestion have been investigated.

**Figure 4:**
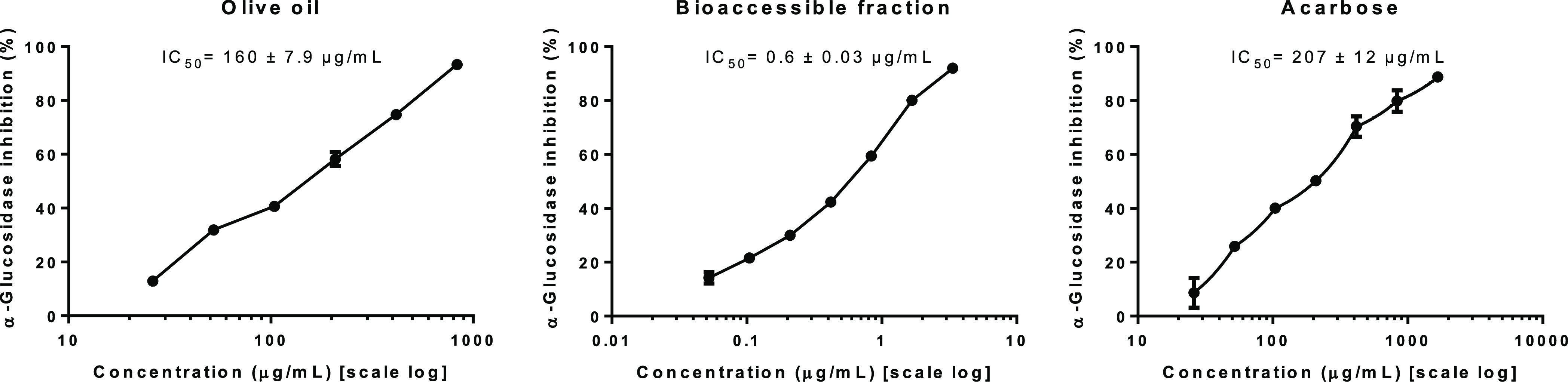
IC_50_ values (μg of dry extract/mL) of the EVOO, the bioaccessible fraction, and the acarbose (positive control, μg/mL) against α-glucosidase enzyme involved in type 2 diabetes.

As is shown in Figure 4, the present study provides evidence of the concentration-dependent inhibitory effect for the Bf on α-glucosidase enzyme. In addition, the conventional antidiabetic drug, namely, acarbose, was used for comparison purposes with the tested extracts. Values of IC_50_ were calculated and displayed in Figure 4 as a measure of their inhibitory potential. The inhibitory IC_50_ value of α-glucosidase in EVOO was 160 ± 7.9 μg of dry extract/mL, suggesting that EVOO phenolics might have a higher enzyme inhibitory activity than acarbose (IC_50_ = 207 ± 12 μg/mL). The IC_50_ value evaluated herein was similar to that obtained for other EVOOs elaborated with “Brava Gallega” variety (IC_50_ = 143–162 μg of dry extract/mL). It should be noted that the studied EVOO was more active than others obtained from “Cornicabra” and “Picual” Spanish varieties (IC_50_ = 246 and 291 μg of dry extract/mL, respectively)^51^ and several Italian varieties (IC_50_ = 184–776 μg of dry extract/mL).^53^ After the *in vitro* digestion, the IC_50_ value was 0.6 ± 0.03 μg of dry extract/mL. This behavior indicated that the *in vitro* digestion positively affected the α-glucosidase inhibition.

The effectiveness of phenolic compounds in inhibiting α-glucosidase activity depends on their mechanism of action, binding affinity, and presence of active sites.^54^ As mentioned above, after the *in vitro* digestion, the instability of the secoiridoids produces the release of simple phenols such as HTy, HTy-Ac, and Ty. In fact, several studies have proved that these compounds are potential antidiabetic substances.^53,55,56^

In addition, flavonoids also showed inhibitory activity on α-glucosidase.^57,58^ Figueiredo-González et al. reported the negative correlation (based on a Pearson correlation test) between Lut and Api and α-glucosidase inhibition in EVOOs.^7^ Therefore, these results suggest that the phenolic compounds present in low concentrations can be more active on the enzyme inhibition than those present in high concentrations, illustrating the high specificity of the phenolic compounds–enzyme interaction. Besides, the possible synergistic or antagonistic effects between all phenolic compounds can also determine the inhibitory activity on this enzyme.^51,59^

This study has made it possible to delve into the transformations that phenolic compounds suffer throughout the gastrointestinal tract. After the oral digestion, the distribution of phenolic compounds between the Wp and the Op was determined by their polarity. After the gastric digestion, extensive secoiridoids hydrolysis was reported. During the intestinal digestion, secoiridoids hydrolysis was again responsible for the release of simple phenols (mainly detected in the Wp), which were stable under the intestinal digestion conditions. The phenolic compounds present in the Op fraction after intestinal digestion (mainly lignans and flavonoids) could interact with the intestinal microbiota to facilitate their degradation and transformation into low-molecular-weight substances potentially absorbable in the colon. The bioaccessible fraction showed the ability to inhibit the α-glucosidase activity to a higher extent than the native EVOO. Nevertheless, future studies are needed to deeply study the promising antidiabetic potential after the *in vitro* digestion and confirm further transformations of these phenolic compounds during the colonic fermentation of the Galician EVOO.

## Supporting Information Available

Quality parameters, sensory attributes, and composition of the studied Galician EVOO (Table S1) (PDF)

## Abbreviations

**ABTS**: 2,2′-azino-bis(3-ethylbenzothiazoline-6-sulfonic acid)
**AC**: antioxidant capacity
**AcH**: acetic acid
**ACN**: acetonitrile
**Ac-Pin**: acetoxypinoresinol
**Api**: apigenin
**Bf**: bioaccessible fraction
**DEA**: dialdehydic form of decarboxymethyl elenolic acid
**Dios**: diosmetin
**DLA**: dialdehydic form of decarboxymethyl ligstroside aglycone (oleocanthal)
**DOA**: dialdehydic form of decarboxymethyl oleuropein aglycone (oleacein)
**DOPAC**: 3,4-dihydroxyphenylacetic acid
**DPPH**: 2,2-diphenyl-1-picrylhydrazyl
**EA**: elenolic acid
**ESI**: electrospray ionization
**EtOH**: ethanol
**EVOO**: extra-virgin olive oil
**Fer**: ferulic acid
**FRAP**: ferric reducing antioxidant power
**GA**: gallic acid
**GAE**: gallic acid equivalents
**HTy**: hydroxytyrosol
**HTy-Ac**: hydroxytyrosol acetate
**Hy-EA**: hydroxy elenolic acid
**IS**: internal standard
**IT**: ion trap
**LC–DAD**: liquid chromatography–diode array detection
**LC-FDL**: liquid chromatography–fluorescence detection
**LC-MS/MS**: liquid chromatography–tandem mass spectrometry
**LC-MS**: liquid chromatography–mass spectrometry
**LDL**: low-density lipoprotein
**Lig**: ligstroside
**LigAgl**: ligstroside aglycone
**LU**: luminescence units
**Lut**: luteolin
**MD**: Mediterranean diet
**MeOH**: methanol
**O-HTy**: oxidized hydroxytyrosol
**OlAgl**: oleuropein aglycone
**Ole**: oleuropein
**Op**: oily phase
***p*****-Cou**: *p*-coumaric
**Pin**: pinoresinol
**PMT**: photomultiplier
**PNP-G**: 4-nitrophenyl α-d-glucopyranoside
**PVDF**: poly(vinylidene difluoride)
**SGD**: simulated *in vitro* gastrointestinal digestion
**SGF**: simulated gastric fluid
**SIF**: simulated intestinal fluid
**SPE**: solid-phase extraction
**SSF**: simulated salivary fluid
**Syr**: syringaresinol
**TE**: Trolox equivalents
**TPC**: total phenolic compounds
**Ty**: tyrosol
**UHPLC/Q-TOF-MS**: ultrahigh-performance liquid chromatography–quadrupole time-of-flight mass spectrometry
**UV–vis**: ultraviolet–visible
**Van**: vanillic acid
**VOO**: virgin olive oil
**Wp**: water phase
