# Supplementary material for: Application of the INFOGEST Standardized Method to Assess the Digestive Stability and Bioaccessibility of Phenolic Compounds from Galician Extra-Virgin Olive Oil
Source: J Agric Food Chem. 2021 Sep 22;69(39):11592–605. doi: 10.1021/acs.jafc.1c04592 (PMC8929668; doi:10.1021/acs.jafc.1c04592)
Supplement: Supplementary file 1 — jf1c04592_si_001.pdf [file jf1c04592_si_001.pdf]

Table S1. Quality parameters, sensory attributes, and composition of the studied Galician EVOO (Supplementary Data).

	**Galician EVOO**	**Regulated values for EVOO (EU Reg 2568/91)**
Quality-related indices		
Free acidity (% oleic acid)	0.26±0.01	≤ 0.8
K_{232}	1.81±0.01	≤ 2.50
K_{270}	0.15±0.01	≤ 0.22
ΔK	< 0.01	≤ 0.01
Peroxide value (meq O_2/kg oil)	5.5±0.2	≤ 20
Sensory analysis		
**Positive attributes**		> 0
Fruity	4.5	
Bitter	3.5	
Pungent	4.2	
**Negative attributes**	0.0	= 0
Genuineness-related indices		
**Fatty acid composition (%m/m methyl esters)**		
Lauric (C12:0)	< 0.10±0.01	
Myristic (C14:0)	0.010±0.001	≤ 0.05
Palmitic (C16:0)	11.12±0.39	7.5-20.0
Palmitoleic (C16:1)	0.82±0.02	0.3-3.5
Margaric (C17:0)	< 0.10±0.01	≤ 0.3
Margaroleic (C17:1)	0.17±0.01	≤ 0.3
Stearic (C18:0)	2.56±0.11	0.5-5.0
Oleic (C18:1)	76.16±1.03	55.0-83.0
Linoleic (C18:2)	7.50±0.48	3.5-21.0
Linolenic (C18:3)	0.69±0.07	≤ 1.0
Arachidic (C20:0)	0.39±0.01	≤ 0.6
Eicosenoic (C20:1)	0.31±0.03	≤ 0.4
Behenic (C22:0)	0.12±0.03	≤ 0.2
Erucic (C22:1)	< 0.10±0.01	
Lignoceric (C24:0)	< 0.10±0.01	≤ 0.2
trans-Oleic isomers C18:1 T	< 0.01±0.01	≤ 0.05
trans-Linoleic + *trans*-Linolenic	0.010±0.001	≤ 0.05
Sterol relative amounts		
Cholesterol (%)	< 0.1±0.1	≤ 0.5
Brassicasterol (%)	< 0.1±0.1	≤ 0.1
Campesterol (%)	2.5±0.2	≤ 4.0
Stigmasterol (%)	1.0±0.1	< Camp.
Apparent β-sitosterol (%)	95.1±3.8	≥ 93.0
Δ^7-Stigmastenol (%)	0.4±0.1	≤ 0.5
Total sterols (mg/kg)	1898±104	≥ 1000
Triterpenic alcohols		
Erythrodiol + uvaol (%)	1.7±0.3	≤ 4.5
Tocopherols (mg/kg)		
α-tocopherol	304.1±0.1	
β-tocopherol	< 0.10±0.01	
γ-tocopherol	< 0.10±0.01	
δ-tocopherol	7.0±0.1	
Total tocopherols	311.1±10.0	
Moisture and volatile matter (%)	0.08±0.02	
Fatty Acid Ethyl Esters (mg/kg)	6±1	
Rancimat (stability time at 100ºC (h))	40.6±1.0	

Values are mean ± standard deviation (n=2).
